# Anterior Minithoracotomy vs. Transcatheter Closure of Patent Ductus Arteriosus in Very Preterm Infants

**DOI:** 10.3389/fped.2021.700284

**Published:** 2021-11-19

**Authors:** Marien Lenoir, Chloé Wanert, Damien Bonnet, Mathilde Méot, Barthélémy Tosello, Virginie Fouilloux, Caroline Ovaert, Sophie Malekzadeh-Milani

**Affiliations:** ^1^Department of Pediatric Cardiac Surgery, Hôpital Timone Enfant, AP-HM, Marseille, France; ^2^Department of Pediatric Cardiology, Hôpital Timone Enfant, AP-HM, Marseille, France; ^3^M3C-Necker, Hôpital Universitaire Necker-Enfants Malades, AP-HP, Paris, France; ^4^University of Paris, Paris, France; ^5^Department of Neonatology, Hôpital Nord, AP-HM, Marseille, France; ^6^Aix-Marseille University, MMG, INSERM, Marseille, France

**Keywords:** transcatheter closure, mini-invasive surgery, prematurity, patent ductus arteriosus, very low birth weight preterm infant

## Abstract

**Introduction:** Patent ductus arteriosus (PDA) is common in preterm infants and contributes to morbidity and mortality. Several studies have shown the feasibility and safety of percutaneous PDA closure. Minimally invasive surgical ligation by anterior thoracotomy is an alternative, bedside technique for PDA closure in very low birth weight preterm infants. Our study aimed to compare short- and medium-term morbidity and mortality between anterior minithoracotomy and transcatheter PDA closure.

**Methods:** From 2010 to 2020, 92 preterm infants <1,600 g underwent PDA closure in two centers: 44 surgical anterior minithoracotomies (center 1) and 48 transcatheter closures (center 2). Using a 1:1 propensity score match analysis, 22 patients in each group were included. The primary outcome was time to extubation after intervention.

**Results:** Preoperative characteristics were similar in both groups after propensity matching (mean weight at procedure, 1,171 ± 183 g; *p* = 0.8). Mean time to extubation was similar: 10 ± 15 days in the surgical group vs. 9 ± 13 days in the transcatheter group (*p* = 0.9). Mean age at hospital discharge was 114 ± 29 days vs. 105 ± 19 days (*p* = 0.2). Two deaths occurred in the surgical group and one in the transcatheter group (*p* = 0.61). Five complications (pneumothorax *n* = 2, chylothorax *n* = 2, phrenic nerve injury *n* = 1) occurred in three patients after surgery. Three complications (chylothorax *n* = 1, endocarditis *n* = 1, renal vein thrombosis *n* = 1) occurred in two patients after percutaneous closure (*p* = 0.63).

**Conclusion:** Equivalent efficiency and safety of surgical mini-invasive vs. transcatheter PDA closure in preterm infants <1,600 g are in favor of applying these alternative techniques according to centers' facilities and competences.

## Introduction

Patent ductus arteriosus (PDA) is very common in preterm newborns and is inversely related to gestational age (weeks) (1). The incidence ranges from 60 to 80% for preterm infants born between 25 and 28 weeks and up to 90% for those born at 24 weeks (1, 2). PDA can result in a significant left-to-right shunting and has been associated with neonatal morbidities: bronchopulmonary dysplasia (BPD), intraventricular hemorrhage (IVH), acute renal failure, necrotizing enterocolitis (NEC), and retinopathy of prematurity (ROP) (1, 3–7). The management of PDA remains controversial. Various treatment options and algorithms are available, but no robust recommendations exist (1, 6). Medical therapy [mostly non-steroidal anti-inflammatory drugs (NSAIDs)] is usually the first-line option, with interventional closure reserved to medical treatment failure or contraindication (7). The current trend is a decrease in surgical treatment.

Surgical PDA ligation was initially described by Gross and consisted in a left thoracotomy with ligation and division of the PDA (8). This technique remains the most frequently performed. In very low weight preterm (VLWB) infants, surgical ligation can be performed through anterior minithoracotomy, in order to minimize postoperative risks. This technique has the advantage of being possibly performed at the bedside. It limits compression and lesions on the left lung and provides a direct view of PDA and vessels (9, 10).

Over the last couple of years, transcatheter PDA closure has emerged as a new alternative (11). Although not yet widely performed in preterm babies, several recent reports have described their efficacy and safety even in VLWB babies (12–14).

In this study, we aimed to assess the short- and medium-term morbidity and mortality of surgical PDA ligation by anterior minithoracotomy, compared to transcatheter PDA closure, in preterm infants weighing <1,600 g.

## Methods

### Population

We retrospectively analyzed data of two referring pediatric cardiology centers dealing with PDA closure in preterm babies. Patients originated from 13 different neonatal intensive care units (NICUs). The surgical group consisted of preterm infants who underwent anterior minithoracotomy ligation at center 1 (Marseille, France) between January 2010 and February 2020. The transcatheter group consisted of preterm infants who underwent percutaneous closure at center 2 (Paris, France) between January 2018 and February 2020. During these periods, there were no transcatheter PDA closures in center 1 (Marseille) and almost no surgical PDA closures in center 2 (Paris).

All preterm newborns with a hemodynamically significant PDA referred for PDA closure and weighing <1,600 g were included. The indication of closure was entirely set by the neonatal teams, usually based on McNamara criteria (15) and after failure of or contraindication to medical treatment. In center 2, transcatheter closure was contraindicated if the anatomy or size of the PDA was not suitable for the insertion of the prosthesis, when the PDA was too short or too large and if adjacent structures were abnormal.

### Procedures

The surgical technique (center 1) has been previously described (9). Most often, bedside ligation was proposed. The procedure was performed under general anesthesia, in supine position with a shoulder roll. A small (1–1.5 cm) horizontal parasternal skin incision was made in the third left intercostal space. After dissection of the intercostal muscle, pericardium was opened and suspended. PDA and left and right pulmonary arteries were exposed, and PDA clipped with one or two metallic vascular clips. Pericardium was closed, and pleural space was deaired.

For the percutaneous procedure, the patient was transferred from the original NICU to center 2 and transferred back at least 24 h after the catheterization, depending on clinical condition. ADO IIAS (Amplatzer^®^, St Jude Medical copyright) devices were used. The Amplatzer Duct Occluder II Additional Sizes (now Piccolo) is contraindicated for infants <700 g and before 3 days' postnatal age. As a rule of thumb, the diameter of the Piccolo device (3, 4, or 5 mm) was chosen by adding 1 mm to the smallest diameter of the PDA, based on echocardiographic dimensions. The length of the PDA was not routinely measured. The shortest length of the Piccolo device (2 mm) was always chosen, to avoid left pulmonary artery or aortic obstruction. The procedure was performed under general anesthesia. Vascular access was obtained through the femoral vein. The procedure was echocardiography- and fluoroscopy-guided, limiting irradiation as much as possible. The technique evolved over time. In the earliest experience, systematic angiographies were performed for PDA measurement with limited amount of contrast. From October 2019, ductal measurements relied solely on echocardiographic assessment. As the center performing surgical closure was not offering transcatheter PDA closure, the patients in the surgical group were not evaluated for percutaneous closure.

### Outcomes

The primary outcome was time to extubation after PDA closure.

We, in addition, investigated survival at discharge without procedural complication; severe morbidity, that is, grades 3–4 IVH (16); cystic PeriVentricular Leukomalacia stages II or III; NEC stage 3 or greater (17); ROP 3 and/or laser treatment (18); and severe BDP, defined as requiring oxygen for at least 28 days in addition to the requirement of 30% or more oxygen and/or mechanical ventilator support or continuous positive airway pressure at 36 weeks' postmenstrual age (19).

### Statistical Analyses

**Figure 1:**
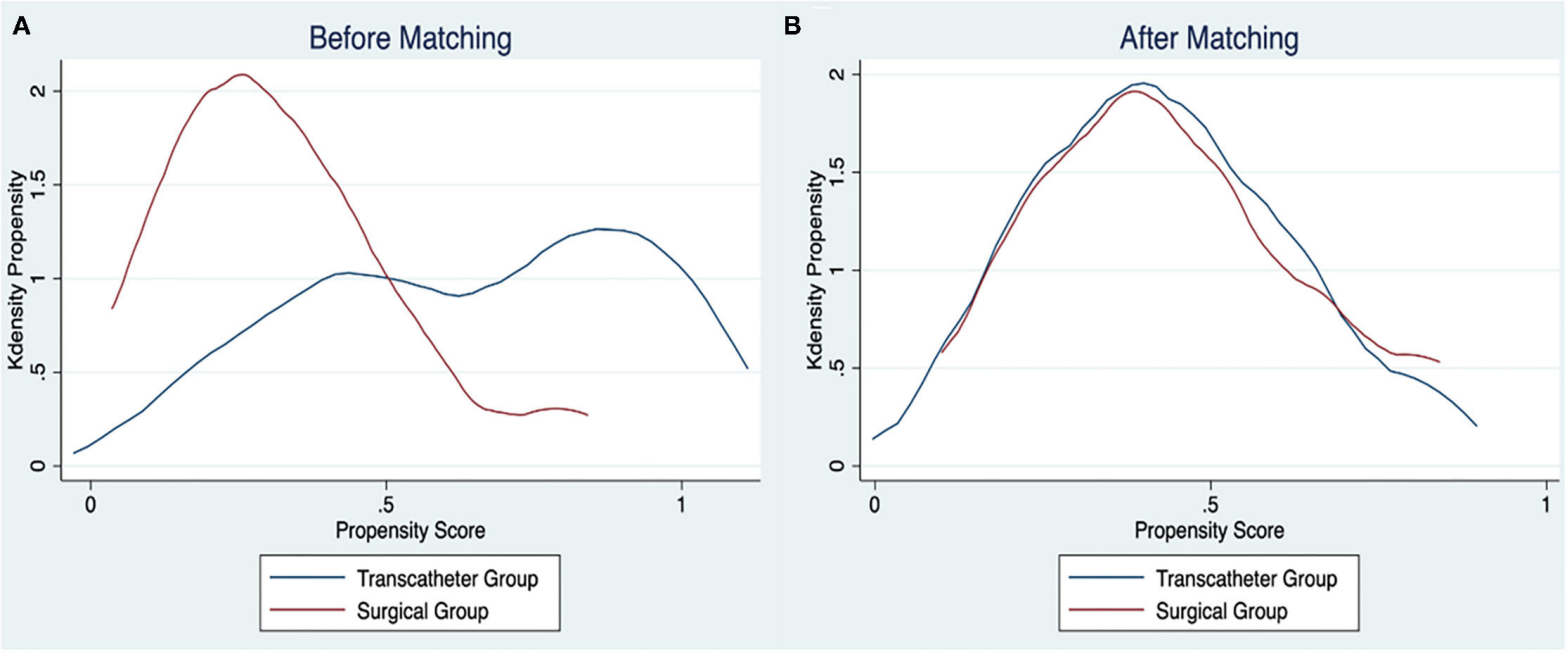
Propensity score distribution. **(A)** Before matching; **(B)** after matching.

Propensity score methodology was used to identify comparable transcatheter and surgical groups. Thirteen variables considered relevant were identified. Therefore, a propensity score was performed to match the transcatheter group patients with surgical group patients (1:1) according to gestational age, sex, weight, corrected age at intervention, associated heart defects, intrauterine growth restriction, NEC, NSAID administration, high-frequency oscillation, invasive or non-invasive ventilation, vasopressive drugs administration, and creatinine level before intervention. Patients were matched using the nearest neighbor method without replacement and using a caliper width of 0.2 of the pooled standard deviation (SD) of the logit of the propensity score. This process yielded 22 well-matched pairs from the 44 surgical cases (50% matched) (Figure 1).

Patients' data were expressed as mean ± SD. Tests for normality were performed (Kolmogorov–Smirnov and Shapiro–Wilk). Only two variables (gestational age and corrected age at intervention) deviated from a normal distribution. There two variables were expressed as median, max, and min. Categorical data are summarized using frequencies and percentages; comparisons were made using the χ^2^-test or Fisher exact test when the number of values was <5. The results were compared, for paired data, using the McNemar test for categorical variables and the Wilcoxon paired signed rank test for quantitative variables. Statistical significance was established as *p* < 0.05. Statistical analyses were performed using STATA statistics (StataCorp LP, College Station, TX).

### Ethics Statement

This study was approved by the local ethics committee (reference 2020-02-04-03).

## Results

### Population

**Figure 2:**
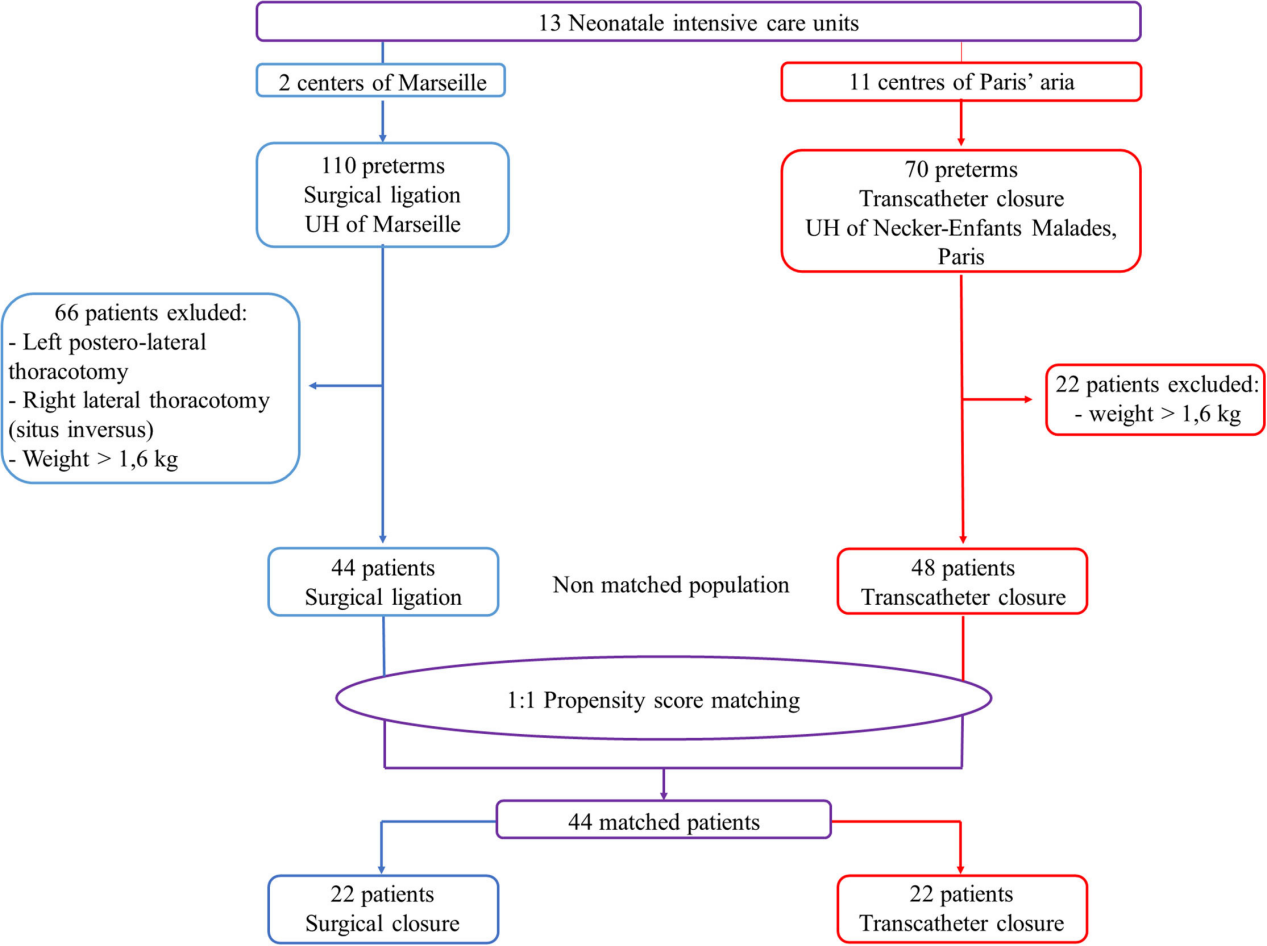
Flowchart of the surgical and transcatheter patients.

Before matching, 44 patients were included in the surgical group and 48 in the transcatheter group (Figure 2). These patients presented significant differences for 13 demographic variables, including age and weight at intervention (Table 1). After matching, two groups of 22 patients each were obtained.

**Table 1:** Patient characteristics before intervention.

	**Before matching**			**After matching**		
	**Transcatheter** **(***n*** = 48)**	**Surgery** **(***n*** = 44)**	***p*** **-value**	**Transcatheter** **(***n*** = 22)**	**Surgery** **(***n*** = 22)**	***p*** **-value**
Female, *n* (%)	24 (50)	24 (54.5)	0.67	11 (50)	11 (50)	1
Gestational age, median (range min–max) (weeks)	25.5 (24–28)	26 (24–29)	**0.024**	25.9 (24–28)	25.5 (24–26.8)	0.5
Birth weight, mean (SD) (g)	751 (119)	756 (143)	0.89	754 (126)	798 (116)	0.22
Birth height, mean (SD) (cm)	32.5 (1.6)	32.5 (2.3)	0.7	32.6 (2)	33.3 (2)	0.38
Antenatal corticosteroid, *n* (%)	41 (85.4)	43 (97.7)	**0.036**	19 (86.4)	21 (95.5)	0.29
IUGR, *n* (%)	3 (6.2)	17 (38.6)	**<0.001**	3 (13.6)	1 (4.5)	0.29
Associated heart defects, *n* (%)	10 (20.8)	14 (31.8)	0.23	5 (22.7)	5 (22.7)	1
PFO/IAC	8 (16.7)	12 (27.3)		5 (22.7)	5 (22.7)	
IVC	1 (2.1)	1 (2.3)		0 (0)	0 (0)	
PVS	1 (2.1)	1 (2.3)		0 (0)	0 (0)	
Hyaline membrane disease, *n* (%)	47 (97.9)	44 (100)	0.34	22 (100)	22 (100)	
IV duration, mean (SD) (d)	21 (12)	18 (10)	0.24	21 (12)	18 (12)	0.66
NIV duration, mean (SD) (d)	12 (13)	8 (11)	**0.06**	10 (11)	11 (14)	0.97
Need for HFO, *n* (%)	34 (70.8)	36 (81.8)	0.22	17 (77.3)	16 (72.7)	0.73
Need for iNO, *n* (%)	12 (25)	14 (31.8)	0.47	4 (18.2)	5 (22.7)	0.71
NEC, *n* (%)	7 (14.6)	5 (11.4)	0.65	2 (9.1)	1 (4.5)	0.56
Fetomaternal infection, *n* (%)	20 (41.7)	35 (79.5)	** <0.001**	10 (45.5)	18 (81.8)	**0.01**
IVH (all grades), *n* (%)	19 (39.6)	10 (22.7)	0.08	8 (36.4)	6 (27.3)	0.52
PDA criteria						
NSAIDs (≥1), *n* (%)	41 (85.4)	40 (90.9)	0.42	19 (86.4)	21 (95.5)	0.29
DA size, mean (SD) (mm) (TTE)	3.1 (0.6)	2.9 (0.8)	0.06	3.2 (0.8)	2.8 (0.6)	**0.03**
Corrected age at intervention (weeks) median (range min–max)	30.5 (26–34)	30.4 (26–43)	0.94	30 (27–33)	29,7 (27–38)	0.4
CA at intervention, mean (SD) (weeks)	30.5 (1.6)	30 (1.7)	0.08	30.4 (1.5)	30.1 (1.6)	0.31
Weight at intervention, mean (SD) (g)	1,191 (203)	1,082 (210)	**0.037**	1,186 (204)	1,157 (165)	0.79
Ventilatory support at intervention, *n* (%)						
IV (CV or HFO)	32 (66.7)	35 (79.5)	0.17	16 (72.7)	16 (72.7)	1
NIV (CPAP or HFNC)	15 (31.2)	8 (18.2)	0.15	6 (27.3)	6 (27.3)	1
SNC	1 (2.1)	1 (2.1)	0.95	0 (0)	0 (0)	
iNO	4 (8.3)	2 (4.5)	0.46	1 (4.5)	1 (4.5)	1
Fio_2_ at intervention, mean (SD) (%)	41 (21)	30 (10)	**0.002**	42 (19)	28 (9)	**0.001**
Vasopressive drugs at intervention, *n* (%)	1 (2.1)	9 (20.5)	**0.005**	1 (4.5)	1 (4.5)	1
Creatinemia at intervention, mean (SD) (μmol/L)	48.6 (30.4)	57 (23.9)	**0.005**	54 (38.1)	51.5 (14.4)	0.24
Hemoglobin at intervention, mean (SD) (g/dL)	11.7 (1.5)	10.3 (1.9)	**0.001**	11.9 (1.7)	9.9 (1.8)	**0.004**
Blood transfusion the day before intervention, *n* (%)	18 (40.9)	21 (47.7)	0.52	7 (36.8)	11 (50)	0.4

*Data are presented as n (%) unless stated differently*.

*IUGR, intrauterine growth restriction; PFO, patent foramen ovale; IAC, interatrial communication; IVC, interventricular communication; PVS, pulmonary valve stenosis; IV, invasive ventilation; NIV, non-invasive ventilation; HFO, high-frequency oscillation; iNO, inhaled nitric oxide; NEC, necrotizing enterocolitis; IVH, intraventricular hemorrhage; NSAIDs, non-steroidal anti-inflammatory drugs; PDA, patent ductus arteriosus; TTE, transthoracic echocardiography; CA, corrected age; Fio_2_, fraction inspired of oxygen. The bold values indicates p ≤ 0.05*.

### Patient Characteristics Before Intervention

Demographic data are shown in Table 1. Mean weeks and birth weight were 25.9 ± 1.1 weeks and 754 ± 126 g, respectively, in the transcatheter group and 25.8 ± 1 weeks (*p* = 0.54) and 798 ± 116 g (*p* = 0.22), respectively, in the surgical group.

At the time of the intervention, mean age, and weight were 31 ± 11 days and 1,186 ± 204 g, respectively, in the transcatheter group and 30.4 ± 1.5 days (*p* = 0.5) and 1,157 ± 165 g (*p* = 0.79), respectively, in the surgical group.

Regarding radiation exposure in the transcatheter group, the median dose area product was 7.7 ± 5.1 μGy/m^2^.

### Outcomes

**Figure 3:**
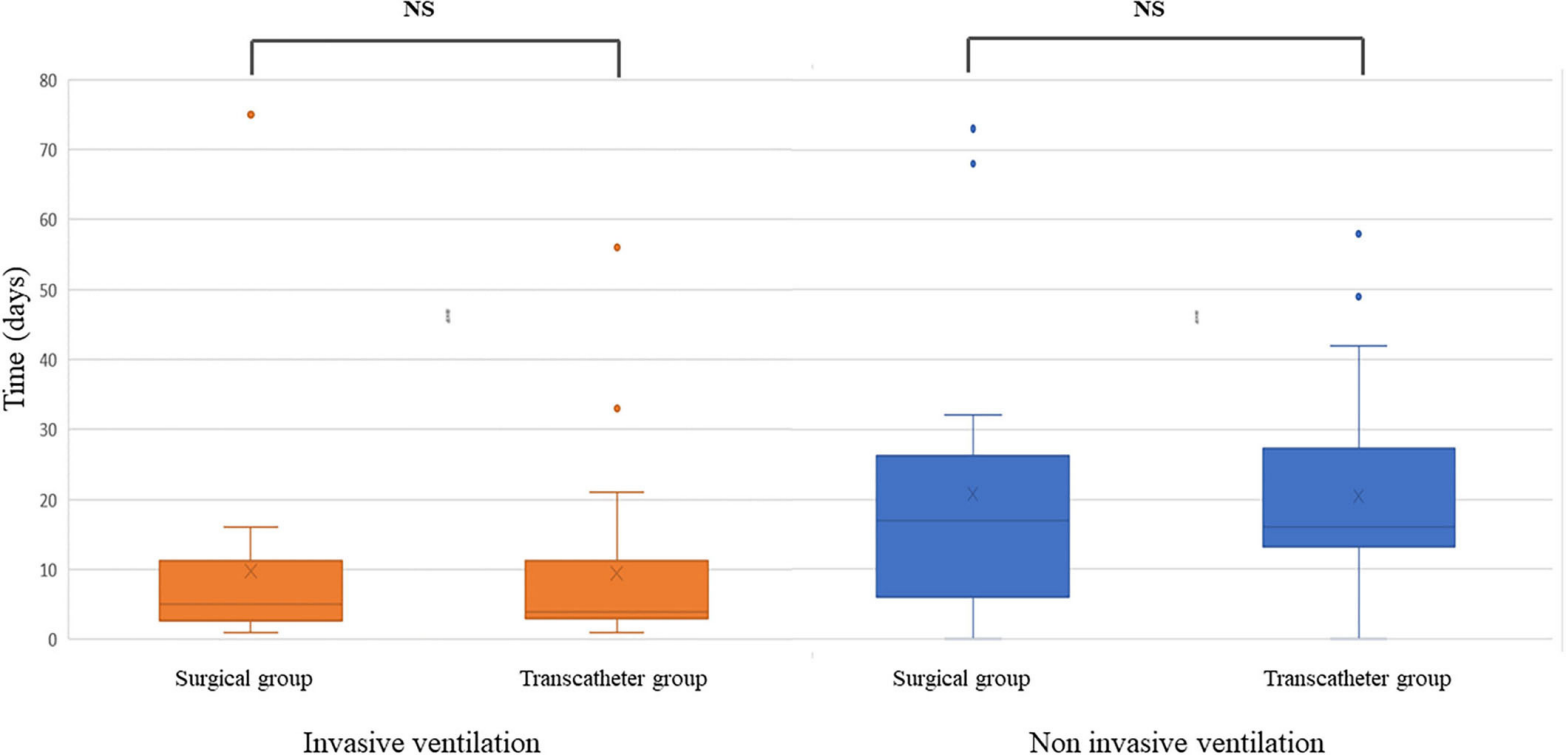
Duration of invasive and non-invasive ventilation after patent ductus arteriosus surgical or percutaneous closure.

Mean time to extubation was similar in both groups: 9 ± 13 days in the transcatheter group and 10 ± 15 days in the surgical group (*p* = 0.89) (Table 2, Figure 3).

**Table 2:** Perioperative outcomes.

	**Transcatheter** **(***n*** = 22)**	**Surgery** **(***n*** = 22)**	***p*** **-value**
Time to extubation (day)	9 (13)	10 (15)	0.88
Total IV duration (day)	28.5 (21.4)	29.9 (22.5)	0.68
Postintervention NIV duration (day)	21 (15)	21 (19)	0.96
Total NIV duration (day)	30 (16.4)	32.2 (18.5)	0.87
Postintervention oxygenotherapy	47 (16)	52 (35)	0.95
duration (day)			
CA at weaning off oxygen (weeks)	36.9 (2.5)	37.2 (4.9)	0.72
Home leave with oxygen, *n* (%)	6 (28.6)	4 (21.1)	0.58
Death, *n* (%)	1 (4.5)	2 (9.1)	
Age at intensive care unit discharge (days)	52 (21)	63 (36)	0.27
Age at hospital discharge (days)	105 (19)	114 (29)	0.24
CA at hospital discharge (weeks)	40.9 (2.4)	42.1 (4)	0.29
Weight at hospital discharge (g)	2,839 (392)	2,860 (752)	0.44

*Data are presented as mean (SD) unless stated differently*.

*IV, invasive ventilation; NIV, non-invasive ventilation; CA, corrected age*.

All procedures succeeded except one in the transcatheter group, because of the creation of an aortic stenosis by the percutaneous device that had to be removed during the same procedure. Two residual shunts (9%) were seen on postprocedure echocardiography the day after intervention in the transcatheter group but disappeared a few days later. No significant left pulmonary artery or aortic stenosis was seen in the two groups. Three complications occurred in 2 of 22 patients (9%) in the transcatheter group: chylothorax (*n* = 1), endocarditis (*n* = 1), and renal vein thrombosis (*n* = 1). Five complications occurred in 3 of 22 patients (14%) in the surgical group: pneumothorax (*n* = 2), chylothorax (*n* = 2), and phrenic nerve injury (*n* = 1) (*p* = 0.63) (Table 3). Post–cardiac ligation syndrome (PCLS) occurred in 2 of 22 patients (9%) of the surgical group vs. none in the transcatheter group (*p* = 0.14). After intervention, the mean creatinine level was similar between both groups (*p* = 0.61). One patient died in the transcatheter group and two in the surgical group. Two patients died because of sepsis not related to the intervention. One patient died of cardiac tamponade 10 days after surgery complicated by hemorrhage of the pulmonary trunk. Mean total invasive ventilation duration was 28.5 ± 21.4 days in the transcatheter group and 29.9 ± 22.5 days in the surgical group (*p* = 0.68), and mean total non-invasive ventilation duration was 31.1 ± 17.3 days (*p* = 0.87) in the two groups. Mean oxygen therapy length and oxygen treatment at discharge were similar between both groups (*p* = 0.96, *p* = 0.58).

**Table 3:** Operative and postoperative outcomes.

	**Transcatheter** **(***n*** = 22)**	**Surgery** **(***n*** = 22)**	***p*** **-value**
Procedural failure	1 (4.5)	0 (0)	0.31
Per procedural complication	0 (0)	2 (9.1)	0.14
PAH	0 (0)	1 (4.5)	
Hemodynamic failure	0 (0)	0 (0)	
Hemorrhage	0 (0)	1 (4.5)	
Patients with postprocedural	2 (9.1)	3 (13.6)	0.63
local complications			
Complications detail:			
PNO	0	2	
Chylothorax	1	2	
Diaphragmatic paralysis	0	1	
Infectious endocarditis	1	0	
Renal vein thrombosis	1	0	
Patients with postprocedural	4 (22)	2 (9)	0.37
general outcomes			
Outcomes details			
PAH	4 (22)	2 (9)	0.37
Hemodynamic failure	0 (0)	2 (9)	0.14
Postligation cardiac syndrome	0 (0)	2 (9)	0.14
Postintervention vasopressor drugs	1 (4.5)	10 (45)	**0.002**
Postintervention TTE			
Residual shunt at the first TTE	2 (9.1)	0 (0)	0.14
Residual shunt 48 h after intervention	0 (0)	0 (0)	
Coarctation of aorta	0 (0)	0 (0)	
Moderate LPA stenosis	1 (4.5)	0 (0)	0.31
Hemoglobin, mean (SD) (g/dL)	11 (1)	12 (2)	0.85

*PAH, Pulmonary Arterial Hypertension; PNO, Pneumothorax; LPA, Left Pulmonary Artery ; RPA, Right Pulmonary artery; TTE, transthoracic echocardiogram; PNO, Pneumothorax; LPA, Left Pulmonary Artery*.

*Data are presented as n (%) unless stated differently. The bold values indicates p ≤ 0.05*.

Half of the patients in each group had secondary sepsis unrelated to the procedure. IVH (any grade) was detected during follow-up in three patients (14%) in the transcatheter group and in two patients (9%) in the surgical group (*p* = 0.63).

Mean age at NICU discharge and mean age at hospital discharge were similar between both groups (*p* = 0.27, *p* = 0.25).

## Discussion

Our study compares transcatheter vs. surgical (anterior minithoracotomy) PDA closure in very small preterm babies. To our knowledge, this is the first series comparing these two techniques.

**Figure 4:**
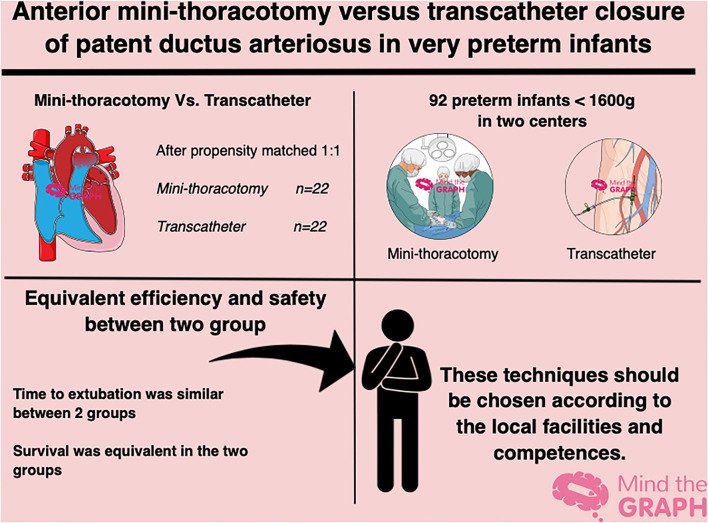
Graphical abstract showing minithoracotomy vs. transcatheter in preterm infants <1,600 g with significant hemodynamic patent ductus arteriosus.

The results of this study are encouraging whatever the technique used, but it is necessary to always consider the least invasive approach possible for these fragile infants (Figure 4), depending on the local skills and settings. We have to be careful not to expose these patients to unnecessary procedures. Indeed, according to Sankar et al. (3), in a recent literature review, early, routine, and widespread treatment to close the PDA in preterm infants does not lead to better outcomes. Some centers even tend toward an expectative approach and prefer to forego surgery after failure of drug treatment (2, 12). Mitra et al. describe a spontaneous closure rate of ~75% at the end of the first year of life in premature infants born <27 weeks, discharged at home with a PDA (20). This debate is not to be resolved in this study, and there is a real cohort of patients who benefit from PDA closure (those born <26 weeks, with chronic respiratory insufficiency or clear signs of hemodynamic repercussions) (3, 21). The indication of PDA closure has to remain in the hands of the neonatologists in charge of the patient.

Surgical ligation through left thoracotomy, as initially described by Gross in 1938 (8), has potential complications, in particular the risks of PCLS (5, 22–24), pneumothorax, chylothorax, vocal cord paralysis, and infection (25, 26). In order to minimize these risks, a less invasive surgical approach can be used, derived from the technique by anterior mini-thoracotomy (10, 21). We believe that this technique is effective and safe for very low birth weight (VLBW) premature infants and limits the adverse effects, in particular due to a reduced operation time (~30 min) and limited intraoperative compressions and lesions of the left lung. However, scientific data are scarce. Karaci et al. (10) reported similar results to ours: in 32 premature infants treated by anterior minithoracotomy with a mean weight of 823 ± 25 g, the mean time to extubation was 13.8 ± 2.3 days.

Transcatheter PDA closure is now possible in VLBW preterm infants, thanks to the modification of the technique avoiding arterial access and thanks to miniaturization of the delivery system and new devices. This procedure remains technically challenging, and although its use is expanding, it remains limited to large and experienced centers (27).

Studies comparing transcatheter to conventional left thoracotomy surgery have shown the non-inferiority of this technique in terms of efficiency and morbidity–mortality (25, 28) and a faster respiratory improvement after procedure (12, 29).

In our study, time to extubation, respiratory outcome with rate of BPD at day 28 and 36 weeks, survival before discharge and morbidity were equivalent in the two groups and similar to those described in other studies (14, 30). Mortality and morbidity, however, were not negligible, probably related to the severity and fragility of the patients included in this study.

Although those two techniques seem to be comparable, they both have their advantages and limitations. Indeed, the main advantage of anterior minithoracotomy technique is that it can be performed at bedside without transfer, but there seemed to be more complications such as pleural lesions and PCLS, although the difference was not significant. PCLS is a classic complication after PDA conventional surgical ligation. Its incidence is variable, ranging from 10 to 45%, and is conversely proportional to gestational age and age and weight at surgery (5, 23, 31). It has not been described after transcatheter PDA closure (4, 14, 28). Furthermore, the need of vasopressive drugs after surgery was more important than after catheterization, consistent with the literature (21).

Percutaneous techniques present a risk, although low, of closure failure and require irradiation of the patient, reduced to a minimum, which averaged 7.7 ± 5.1 μGy/m^2^ in our population. Since October 2019, all percutaneous closures were done without angiography. Transthoracic echocardiogram plays indeed a key role in transcatheter PDA closure in preterm infants. With increasing experience of the sonographer, the morphology and dimensions of the ductus, the adequate placement of the device, the presence of residual shunting, and the absence of aortic or left PA obstruction are better appreciated by ultrasound (two-dimensional imaging and Doppler) than by angiography. Piatek et al. (32) described a case of contrast-induced hypothyroidism after percutaneous PDA device closure in VLBW preterm infants. This is one more reason to avoid angiography. In our study, one percutaneous closure failed, because the device caused an aortic coarctation and had to be removed. The PDA was nevertheless closed on the follow-up echocardiography the next day. Other potential complications may occur such as pulmonary artery stenosis, device embolization, and tricuspid regurgitation (5, 13, 30) but were not observed in our matched population. In our study, patients did not have systematic echo Doppler of vascular access after procedure, so that the rate of vascular thrombosis could not be assessed.

### Limitations

The major limitations to our study are the retrospective observational analysis and the presence of a bicentric cohort with each of the centers using only one technique. The management of preintervention and postintervention may have been different in the 13 NICUs in charge of the patients, in particular with regard to extubation criteria. We used a propensity score matching to minimize differences in characteristics before PDA closure. This resulted in a relatively small sample size, which may influence the power of some analyses and increase the risk of type II statistical error. In addition, despite matching, we were not able to capture all criteria related to prematurity, those specific to the practitioner, and to the procedure that may have played a role in preinterventional and peri-interventional care and influenced clinical outcomes. Evolution in preintervention and postintervention care over time needs to be considered, as well as the effects of the learning curve.

In addition, the period for inclusion of anterior minithoracotomy–treated infants spans 10 years, making it likely that other treatments and routines have changed during this time.

Further analyses are needed to study the long-term evolution of these premature newborns and to determine the impact on cardiac function and especially the size of pulmonary arteries and descending aorta.

In conclusion, mini–anterior thoracotomy PDA ligation and percutaneous PDA closure are two equivalent techniques that are effective, safe, and reproducible for preterm infants <1,600 g with significant hemodynamic PDA. Local setting and facilities as well as technical expertise should drive choice of one vs. the other.

## Data Availability Statement

The raw data supporting the conclusions of this article will be made available by the authors, without undue reservation.

## Ethics Statement

The studies involving human participants were reviewed and approved by (Committee Ethics of Aix-Marseille University) (reference 2020-02-04-03). Written informed consent to participate in this study was provided by the participants' legal guardian/next of kin.

## Author Contributions

All authors listed have made a substantial, direct, and intellectual contribution to the work and approved it for publication.

## Conflict of Interest

The authors declare that the research was conducted in the absence of any commercial or financial relationships that could be construed as a potential conflict of interest.

## Publisher's Note

All claims expressed in this article are solely those of the authors and do not necessarily represent those of their affiliated organizations, or those of the publisher, the editors and the reviewers. Any product that may be evaluated in this article, or claim that may be made by its manufacturer, is not guaranteed or endorsed by the publisher.
